# Special Issue “Biomechanical Analysis in Physical Activity and Sports”

**DOI:** 10.3390/jfmk10020116

**Published:** 2025-03-30

**Authors:** Pedro Forte

**Affiliations:** 1 Department of Sports, Higher Institute of Educational Sciences of the Douro, 4560-708 Penafiel, Portugal; pedromiguel.forte@gmail.com or pedromiguel.forte@iscedouro.pt; 2 Department of Sports Sciences, Instituto Politécnico de Bragança, 5300-253 Bragança, Portugal; 3 Research Center for Active Living and Wellbeing, Instituto Politécnico de Bragança, 5300-253 Bragança, Portugal

## 1. Introduction

Biomechanics plays a vital role in helping us understand how the human body moves, especially in the context of sports and physical activity. By applying principles from physics and engineering, biomechanical analysis allows us to study the forces acting on the body. This is incredibly valuable not only for enhancing athletic performance but also for health and physical activity-related analysis. Recent technological advances, including motion capture systems, force plates, electromyography (EMG), and computational fluid dynamics, have provided us with powerful tools for measuring and modeling movement with unparalleled precision [1,2,3]. These technologies have been adopted across various sports, including running, cycling, and swimming, helping athletes and coaches in enhancing their performance techniques [2,3]. In addition, the emergence of wearable technology and artificial intelligence (AI) has further advanced real-time analysis capabilities. Today, athletes can receive immediate feedback through sensors embedded in their gear, allowing them to adjust their technique on the spot, which also plays a big part in preventing injuries [1].

Despite significant advances in sports biomechanics, several key gaps remain to be addressed. One of the major challenges is translating findings from controlled laboratory environments into real-world sports settings. While many studies have contributed to our understanding, they do not always reflect the complex, dynamic conditions of actual performance [4,5]. To overcome these challenges, there is a need for more field-based research that integrates biomechanical assessments into both training and competitive environments [5]. Another limitation is the narrow focus on elite athletes. Research often overlooks recreational athletes, children, older adults, and individuals with disabilities [6]. It is essential to expand our studies to include these groups to develop more inclusive and broadly applicable training programs [7]. Furthermore, while many biomechanical interventions show immediate benefits, there is a need for long-term studies that evaluate how these changes influence injury risk and athletic development over time [1,4].

In practical terms, the applications of biomechanics are vast. By examining joint angles, muscle activation, and force distribution, biomechanists and healthcare professionals can identify inefficiencies in movement and develop personalized training plans [8]. For instance, runners can benefit from gait retraining programs that not only improve efficiency but also help prevent common overuse injuries. Furthermore, insights from biomechanics inform rehabilitation processes, ensuring that injured athletes return to sport safely and effectively [4]. Additionally, this area plays a crucial role in improving sports equipment design. Whether it is running shoes or adaptive equipment for athletes with disabilities, biomechanics ensures that these tools are optimized for comfort and performance [2]. Finally, coaches are increasingly leveraging biomechanical data to personalize exercises and improve techniques, addressing each athlete’s specific strengths and areas for improvement [9].

Looking ahead, the future of biomechanics lies at the intersection of technology and interdisciplinary collaboration. AI-powered models are set to enhance how we analyze human movement, facilitating the delivery of personalized training and immediate feedback [2]. We also anticipate the emergence of more portable and user-friendly wearable sensors, enabling high-quality biomechanical assessments in real-world settings and enhancing the applicability of research findings to actual conditions [10].

In addition, exploring how the nervous system interacts with biomechanics may result in more effective rehabilitation strategies, especially for athletes recovering from neurological injuries [11]. This approach will help ensure biomechanics contributes to a more inclusive and forward-thinking sporting environment [12].

The recently published Special Issue, “*Biomechanical Analysis in Physical Activity and Sports*”, brings attention to the exciting ways biomechanics shapes the future of sports and exercise. As new technologies facilitate the study of bodily movements, there is an increasing need to connect research with real-world applications in sports, fitness, or athletics. This Special Issue allows researchers, coaches, and sports professionals to share novel insights, practical solutions, and creative collaborations that enhance athlete performance, safety, and training effectiveness. It presents a valuable chance to explore the impact of biomechanics on movement and competitive performance.

## 2. Biomechanical Analysis in Physical Activity and Sports

This Special Issue on Biomechanical Analysis in Physical Activity and Sports presents a comprehensive collection of studies that collectively contribute to a more applied understanding of human movement in sports performance and clinical contexts. The research published here spans several key thematic areas, including neuromuscular function, gait analysis, anthropometry, sport-specific techniques, motor learning, and methodological innovation, each offering relevant contributions to the scientific community and practitioners working in the field.

Biomechanical Analysis in Physical Activity and Sports provides valuable insights into age-related changes in neuromuscular control and gait patterns to rehabilitation practices, helping to refine strategies for patients recovering from surgery and anatomic pathologies, improving their mobility and quality of life [13]. Finally, it offers a novel approach to assessing speed fluctuation in swimming, which is integral for performance optimization in aquatic sports, providing athletes and coaches with valuable insights into pacing strategies [14].

The contributions to this Special Issue reflect the multidimensional nature of biomechanical analysis in sport and physical activity. The integration of new technologies, interdisciplinary methods, and population-specific considerations showcases the maturity and continued evolution of the field. These papers deepen the scientific understanding of movement and offer practical applications for coaches, clinicians, and practitioners seeking to enhance performance and wellbeing across diverse populations.
